# Molecularly targeted therapies in cancer: a guide for the nuclear medicine physician

**DOI:** 10.1007/s00259-017-3695-3

**Published:** 2017-04-10

**Authors:** S. Lheureux, C. Denoyelle, P. S. Ohashi, J. S. De Bono, F. M. Mottaghy

**Affiliations:** 1 Drug Development Program, Medical Oncology Princess Margaret Cancer Centre, Toronto, Canada; 2 Normandie Univ, UNICAEN, Inserm, ANTICIPE (Interdisciplinary Research for Cancers Prevention and Treatment, axis 2 BioTICLA (Biology and Innovative Therapeutics for Locally Aggressive Cancers), 14000 Caen, France; 3 Comprehensive Cancer Center François Baclesse, UNICANCER, 14076 Caen, France; 4 Department of Medical Biophysics, Department of Immunology, Campbell Family Institute for Breast Cancer Research, Princess Margaret Cancer Centre, University of Toronto, Toronto, Canada; 5 Drug Development Unit, The Institute of Cancer Research & The Royal Marsden NHS Trust, Sutton, UK; 6 Department of Radiology and Nuclear Medicine, Maastricht University Medical Centre (MUMC+), Postbox 5800, 6202 AZ Maastricht, The Netherlands; 7 Department of Nuclear Medicine, University Hospital, RWTH Aachen University, Pauwelsstr. 31, 52072 Aachen, Germany

**Keywords:** targeted therapies, pathway, imaging, cancer

## Abstract

Molecular imaging continues to influence every aspect of cancer care including detection, diagnosis, staging and therapy response assessment. Recent advances in the understanding of cancer biology have prompted the introduction of new targeted therapy approaches. Precision medicine in oncology has led to rapid advances and novel approaches optimizing the use of imaging modalities in cancer care, research and development. This article focuses on the concept of targeted therapy in cancer and the challenges that exist for molecular imaging in cancer care.

## Targets in Cancer: Opportunities for Treatment Innovation

Extensive investigations of carcinogenesis and tumor characterization have identified various deregulations within tumors and their microenvironments and have helped steer the direction of drug development in cancer [1, 2]. Target engagement can be achieved through several modalities that modulate or interact with cell surface receptors (monoclonal antibodies), intracellular cascade pathways and signaling (small molecule tyrosine kinase inhibitors) or micro-environment effects related to tumor vasculature or hypoxia. There have also been interesting results leveraging antibody-drug conjugates to increase cytotoxic drug delivery [3]. Modulating the immune environment by way of promoting dynamic changes in cancer cell interaction with immune cells is a very active area of study, including cellular therapy using *ex-vivo* propagation of immune cells, vaccines and checkpoint inhibitors [4]. Finally, improved delivery of targeted agents to cancer cells using nano-particles such as porphysomes presents tremendous opportunity to precision-bomb cancer cells and reduce bystander or collateral toxicity [5]. These biological abnormalities have already driven and will further enhance innovation of probes for molecular imaging beyond FDG-PET imaging [6–8].

Aberrations in various cellular signaling pathways are instrumental in regulating cellular metabolism, tumor development, growth, proliferation, metastasis, and cytoskeletal reorganization [9]. Therefore an improved understanding of the pathway is requisite to evaluate the impact of a potential drug target and the associated imaging assessment for response. We will describe the main pathways currently targeted in cancer care and how these new treatment options may impact when and how molecular imaging can be used.

## Cancer Cell Signaling Pathways – Fig. 1

### Targeting PI3K/AKT/mTOR signaling in cancer

The phosphatidylinositol 3-kinase (PI3K)/AKT/mammalian target of rapamycin (mTOR) pathway plays a critical role in the malignant transformation of human tumors and their subsequent growth, proliferation, and metastasis [10]. The PI3K/AKT/mTOR signaling pathway regulates central aspects of cancer biology such as metabolism (e.g. increased activation of the GLUT transporters), cellular growth, and survival [11]. Upon stimulation of receptor tyrosine kinases, PI3K phosphorylates phosphatidylinositol-4,5-bis-phosphate 2 (PIP2) into PIP3 resulting in the activation of AKT. Among its targets, AKT controls the activation of the downstream pathway effector, the mammalian target of rapamycin (mTOR), which activates two key substrates 4EBP1 and p70S6K. This results in increased translation of target genes involved in angiogenesis (VEGF) and cell cycle progression (cyclin D1, c-Myc). The primary negative regulator of the PI3K pathway is the tumor suppressor phosphatase and tensin homolog (PTEN). PTEN can dephosphorylate PIP3, reversing AKT activation and inhibiting further downstream signaling; however, in the absence of PTEN inhibition, AKT phosphorylates and leads to mTOR activation [12]. Various activating mutations in oncogenes together with the inactivation of tumor suppressor genes are found in diverse malignancies across almost all members of the pathway [9]. Substantial progress in uncovering PI3K/AKT/mTOR alterations and their roles in tumorigenesis have enabled the development of novel targeted molecules and, alongside this, the potential for developing efficacious anticancer treatment. Two approved anticancer drugs, everolimus and temsirolimus, exemplify targeted inhibition of PI3K/AKT/mTOR in the clinic and many others are in development for many different types of cancer. Adverse events observed in patients treated with mTOR inhibitors are fairly consistent, irrespective of each specific indication. They include cutaneous and mucosal events (i.e., stomatitis and skin rash), pulmonary dysfunction (non-infectious pneumonitis), metabolic abnormalities (elevated blood levels of glucose, cholesterol, and triglycerides), as well as immune-related events (i.e., increased incidence of infections) [13]. As far as the risk of infections is concerned, mTOR inhibitors were first developed as immune suppressive agents and are still widely used as such in the transplantation setting. Metabolic and immune-related adverse events are on-target effects of mTOR inhibition, while cutaneous and mucosal effects may have a less direct association with mTOR inhibition [13]. With the goal of being more selective and potent, PI3K and Akt inhibitors have been developed and are under evaluation. Several preclinical studies have systematically investigated the value of FDG or FET PET imaging. Both tracers seem to be valuable biomarkers for the prediction and measuring of response [14–16]. In a first clinical study the value of a specifically VEGF targeting ligand has been evaluated and the results seem to be promising [17].

**Fig. 1:**
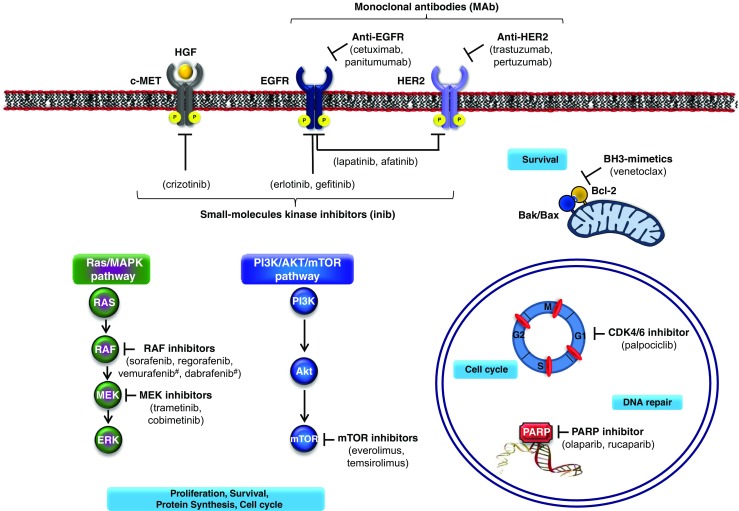
Therapeutic strategies for targeting cancer cells. This figure summarizes the most relevant drivers and signaling cascades involved in solid tumors and treatments that are currently in clinical use (except for venetoclax used for the treatment of CLL). ^#^BRAF inhibitors. Abbreviations: Akt: AKR mouse thymoma kinase; Bak, bcl-2 antagonist killer 1; Bax, Bcl-2 associated X protein; Bcl-2, B-cell lymphoma gene 2; CDK, cyclin-dependent kinase, EGFR (ErbB1), epidermal growth factor receptor; CLL, chronic lymphocytic leukemia; ERK, extracellular signal-related kinase; FGFR, fibroblast growth factor receptor; HER2 (ErbB2), human epidermal growth factor receptor 2; HGF, Hepatocyte growth factor; mTOR, mammalian target of rapamycin; MAPK, mitogen-activated protein kinase; MEK, MAPK/ERK kinase; PARP, poly(ADP-ribose) polymerase; PI3K, phosphoinositide 3-kinase; RAF (RAF1, v-raf-1 murine leukemia viral oncogene homolog 1 and BRAF, v-raf murine sarcoma viral oncogene homolog B1)

### The MAPK Pathway

Mitogen-activated protein kinase (MAPK) cascade is a critical pathway for human cancer cell survival, dissemination, and resistance to drug therapy. One of the most studied MAPK pathways is the extracellular signal-regulated kinase (ERK) pathway. ERK is a subgroup of MAPKs that is activated by external factors such as growth factors and mitogens [18]. The MAPK/ERK pathway is activated by upstream genomic events and/or activation of multiple signaling events where information coalesces at this important nodal pathway point. This pathway is tightly regulated under normal conditions by phosphatases and bidirectional communication with other pathways, such as the AKT/mTOR pathway [19]. Ligand-mediated activation of receptor tyrosine kinases such as Epidermal Growth Factor Receptor (EGFR) initiate the cascade of ERK signaling that flows through RAS GTPase, which acts as a molecular on/off switch. Once RAS, a family of various oncogenes such as *KRAS*, *NRAS* and *HRAS*, is turned on it recruits and activates proteins necessary for the propagation of growth factor and other receptor signals, including RAF and PI3K. RAF activation is achieved through a complex process that requires lipid and protein binding, conformational changes, and regulatory phosphorylation and dephosphorylation events. There are three RAF proteins in mammals, including BRAF, and they can all activate MAP kinase kinase (MEK) just upstream of ERK [20]. The downstream MAPK/ERK signaling node, predominantly activated by upstream SRC/RAS/RAF signaling, is also regulated by modulation through parallel pathways. Several mutations involving the MAPK/ERK pathway have been identified, occurring upstream in membrane receptor genes (EGFR), in signal transducers (RAS), and in downstream kinases belonging to the MAPK/ERK pathway itself (BRAF) in human cancers and are ripe for targeting [19, 21]. Currently approved for the treatment of unresectable metastatic melanoma with somatic point mutation in the *BRAF* gene resulting in constitutive activation of BRAF (V600E) kinase, inhibitors of B-RAF kinase are being studied alone (vemurafenib, dabrafenib) and in combination with inhibitors of MEK (cobimetinib) and other pathways to optimize treatment of many tumor types. This therapeutic is well tolerated with the most common adverse effects including skin reactions, photosensitivity, headache and arthralgia, although an increased risk of development of localized cutaneous squamous cell carcinoma has also been observed [22, 23]. Inhibitor of MEK in monotherapy (Trametinib) was also shown to be effective for the treatment of patients with un-resectable or metastatic melanoma harboring activating BRAF V^600E/K^ mutations [24]. However, therapies targeted toward MAPK/ERK components have variable response rates when used in different solid tumors, such as colorectal cancer and ovarian cancer. Understanding the differential nature of activation of the MAPK/ERK pathway in each tumor type is critical in developing single and combination regimens [19]. Several studies have evaluated development of new tracers to specifically target the altered pathways [25–34]. Also, standard FDG PET still has its role for therapy evaluation or prediction of response [35].

### HGF/c-MET/Pathway

The Met receptor tyrosine kinase is the prototypic member of a small subfamily of growth factor receptors that when activated induce mitogenic, motogenic, and morphogenic cellular responses. MET gene encodes the receptor tyrosine kinase (RTK) MET or c-MET that is activated by the ligand HGF (hepatocyte growth factor). Abnormal Met signaling has been strongly implicated in tumorigenesis, particularly in the development of invasive and metastatic phenotypes [36]. The HGF-MET binding leads to MET phosphorylation and subsequent activation of different effectors such as GRB2, CRK, CRKL, SHC and GAB1. These effectors trigger the activation of other pathways including RAS-MAP (through interaction between SOS and GRB2), PI3K-AKT, STAT3 and NF-kB [37]. The MET pathway is maintained by a balance between stimulating signals (PAX5, PAX8 and HIFα) and down regulation mechanisms such as ubiquitination mediated by CBL or cleavage by metalloprotease ADAM-like and gamma-secretase. Deregulation of the HGF-MET cellular axis in cancer can be detected at different molecular levels such as by changes in extent of protein expression, by variation in gene copy number and by presence of gene mutations. Hyperactivation of this pathway occurs in different cancers and is related to a worse prognosis [38]. A small molecule inhibitor (crizotinib) was approved for the treatment of patients with metastatic non-small cell lung cancer (NSCLC) whose tumors are anaplastic lymphoma kinase (ALK)-positive as detected by an FDA-approved test [39]. A recent preclinical study evaluated the potential of FLT in crizotinib treatment [40]. Numerous clinical trials with new monoclonal antibodies or tyrosine kinase inhibitors targeting MET are ongoing in different solid tumors. Radiolabelling of these monoclonal antibodies for *in vivo* assessment of the biodistribution using the radionuclides Copper-64 or Zirconium-89 (both positron emitters with long half-life; 12.8 and 3.3 days, respectively) are interesting new possibilities for individualized therapy concepts [41–45].

### HER Pathway

The ERBB family of receptor tyrosine kinases has a central role in the tumorigenesis of many types of solid tumors [46]. The ErbB receptor tyrosine kinase family consists of four cell surface receptors: ErbB1/EGFR/HER1, ErbB2/HER2, ErbB3/HER3, and ErbB4/HER4. ErbB receptors are typical cell membrane receptor tyrosine kinases that are activated following ligand binding, except ErbB2, and receptor dimerization. ERBB family receptors activate several downstream pathways, including the RAS–ERK and PI3K–AKT pathways [47]. Inappropriate activation of EGFR and ERBB2 in cancer can occur through a range of mechanisms, including overexpression (often due to gene amplification), point mutations, partial deletions and autocrine ligand–receptor stimulation [48]. The frequent activation of ERBB family members in cancer makes them attractive therapeutic targets and various members have been approved for the treatment of several cancers. Trastuzumab is a monoclonal antibody given intravenously that interferes with the HER2/neu receptor, and is well known for its activity in ErbB2-positive breast cancer [49, 50]. Another HER2 antibody (pertuzumab) was also approved for use in combination with trastuzumab and docetaxel for neoadjuvant treatment of ErbB2-positive breast cancer patients [51]. Lapatinib is the first dual inhibitor in clinical use acting as a tyrosine kinase inhibitor of EGFR and HER2 used in the treatment of ErbB2-overexpressing breast cancer [52]. Gefitinib, erlotinib, and afatinib are orally active inhibitors of EGFR tyrosine kinase activity that are used in the treatment of ERBB1-mutant lung cancer [53]. Cetuximab and panitumumab are monoclonal antibodies that target ErbB1 and are used in the treatment of colorectal cancer [54, 55]. Recently several labeling concepts and first preclinical as well as clinical studies of specific tracers based on the above mentioned ligands have been presented and display very promising results that will have to be further translated into the clinical setting [43, 44, 56–63].

### DNA Damage Response Processes – Fig. 1

Tumor initiation and progression is inexorably linked to disruption of the DNA damage-response (DDR) [64]. A novel therapeutic strategy, cellular DDR processes engage various proteins that sense DNA damage, initiate signaling pathways to promote cell-cycle checkpoint activation, trigger apoptosis, and coordinate DNA repair [65].

- DNA Repair

The DNA repair pathway is a complex set of cellular responses that are elicited following DNA damage, commonly including base and sugar modifications, single- and double-strand breaks, DNA-protein cross-links, and base-free sites [64]. To counteract these damages, multiple DNA repair pathways exist with subpathways providing lesion specificity. These processes include base excision repair, mismatch repair, nucleotide excision repair, and double-strand break repair (DSBs), which comprise both homologous recombination and non-homologous end-joining.

An underlying hallmark of cancers is their genomic instability, which is associated with a greater propensity to accumulate DNA damage. Historical treatment of cancer by radiotherapy and DNA-damaging chemotherapy is based on this principle, yet it is accompanied by significant collateral damage to normal tissue and unwanted side effects. Targeted therapy based on inhibiting the DDR in cancers offers the potential for a greater therapeutic window by tailoring treatment to patients with tumors lacking specific DDR functions. The recent approval of olaparib (Lynparza), the poly (ADP-ribose) polymerase (PARP) inhibitor for treating tumors harboring *BRCA1* or *BRCA2* mutations, represents the first medicine based on this principle, exploiting the synthetically lethal genetic relationship underlying in the tumor [66, 67]. Olaparib has been the first PARP inhibitor approved in practice; and many others are in the pipeline with the main side effects being nausea/vomiting, hematologic and fatigue [68]. There is an increasing body of evidence indicating benefit of targeting pathways involved in maintaining DNA integrity, beyond BRCA1 and BRCA2 signaling [65, 69] with the ability to leverage deficiencies in homologous recombination sharing phenotypic features of those tumors exhibiting BRCA-like behavior (BRCAness/HRD phenotype) [70]. Cancer-specific defects in DNA repair pathways can be used as targets for personalized therapeutic approaches [71, 72]. Specific *in vivo* imaging of PARP activity has been successfully demonstrated, whether this approach will be translatable to the clinical situation remains to be shown [73, 74]. There are also further concepts to develop PET and SPECT radiotracers that are related to the mechanisms of action of new drugs such as using imaging to evaluate DNA damage repair proteins [8].

However, DNA repair pathways can enable tumor cells to survive DNA damage that is induced by chemotherapeutic treatments; therefore, inhibitors of specific DNA repair pathways might prove efficacious when used in combination with DNA-damaging chemotherapeutic drugs [75]. Mechanisms of resistance to standard chemotherapy or targeted therapy are an active area of research.

- Cell Cycle Checkpoint

The cell cycle is a complex process involving numerous regulatory proteins, of which the cyclin-dependent kinases (CDK) are central. These proteins regulate a cell’s progression through the stages of the cell cycle and are, in turn, regulated by numerous proteins, including p53, p21, p16, and cdc25. Downstream targets of cyclin-CDK complexes include pRb and E2F [76]. The cell cycle is altered in cancer due to alterations either in oncogenes that indirectly affect the cell cycle or in tumor suppressor genes or oncogenes that directly impact cell cycle regulation, such as p53, p16, or viral infection including Human Papillomavirus (HPV). Tumor-associated cell cycle defects are often mediated by alterations in CDK activity [77]. It has become progressively clear that cancer cells have defective cell cycle checkpoints. These defects, which very likely contribute to neoplastic transformation and progression by increasing genetic instability, can be exploited to envision strategies that will increase our treatment options against cancer [78]. The inhibition of the checkpoint kinases can be achieved with conventional DNA damaging therapies. In this case cancer cells lacking the G1 checkpoint lose the remaining protective effect of the G2/M checkpoint and die by mitotic catastrophe [79]. The other strategy relies on addiction of cancer cells transformed by active oncogenes (such as Ras, Myc or Cyclin E) to ATR, Chk1, and Wee1 kinases that allow them to cope with a high level of replication stress [79]. Several agents are currently in development. An earlier study was able to demonstrate *in vivo* CDK4/6 inhibition by means of FDG and FLT [80]. Specific labeling of the new agents in development could evaluate the theranostic potential.

### The Retinoblastoma Pathway

Retinoblastoma (Rb) protein is a regulator of G1/S checkpoint. When Rb is not phosphorylated it binds and represses the transcription factor E2F; but when RB is phosphorylated by a cyclin-dependent-kinase (CDK4 and CDK6), E2F is released and DNA replication is active [81]. Different types of cyclin D associate with CDK4 or CDK6, creating a complex able to phosphorylate Rb. CDK4/6 activity is inhibited by INK4 family proteins, such as p16^Ink4a^ [82]. Dysregulated activation of the cyclin D-CDK4/6-INK4Rb pathway is frequently observed in a range of tumor types. Different types of genomic mutations have been reported: amplification in *CCDN1* (the gene that encodes cyclin D1), amplification of *CDK4* or *CDK6*, mutation in *CDKN2A* (genes that encode p16Ink4a) or activating aberrations in PI3K/AKT/mTOR or RAS/RAF/MEK [78]. Activity of CDK4/6 may be targeted by specific agents and clinical trials are ongoing with CDK4/6 inhibitors like palbociclib, ribociclib and abemaciclib. The main toxicities reported are hematological and fatigue [83]. In breast cancer palpociclib has already been approved in the United States as first-line treatment in association with letrozole in postmenopausal women with ER-positive and HER2-negative metastatic breast cancer [84]. The value of FDG for therapy response assessment has been demonstrated [85], however specific labeling of the inhibitors might also be of interest in this setting.

### P53

*TP53* is one of the most important tumor suppressor genes and is frequently mutated in human cancers [86]. P53 has different functions including activation of DNA repair proteins, G1/S cell cycle checkpoint allowing cells to fix the DNA damage and activation of apoptosis. Generally, p53 functions as a transcription factor that is stabilized and activated by various genotoxic and cellular stress signals, such as DNA damage, hypoxia, oncogene activation and nutrient deprivation, consequently leading to cell cycle arrest, apoptosis, senescence and metabolic adaptation [87]. *TP53* somatic mutations are a defining event in cancers [88–90]. When p53 is not functioning, cells are dependent on S/G2 checkpoint to arrest growth and allow DNA repair. Inhibition of S/G2 checkpoint in p53 deficient tumor cells results in the favoring of apoptosis and enhancement of the chemotherapy effect. Wee1 is a tyrosine kinase implicated in the G2 checkpoint and its inhibition in HGSOC is under investigation in various clinical trials. P53 seems an attractive target in cancers and contemporary strategies targeting p53 have been developed, including gene therapy to restore p53 function, inhibition of p53-MDM2 interaction, restoration of mutant p53 to wild-type p53 or targeting p53 family proteins; however, p53-targeted therapy remains challenging [87, 91, 92]. Different types of *TP53* mutations have been described but the functionality of this mutation is complex [93]. Some types of *TP53* mutations are termed gain-of-function or loss-of-function mutations and the exact impact on patient outcome and response to treatment is not well established; investigations remain on-going. The efficient implementation of p53-targeting treatments into clinical practice requires thorough understanding of the mechanisms governing p53 response in cancer cells [94].

- Programmed Cell Death

Apoptosis, autophagy and programmed necrosis are mediated by an intracellular program, which is deregulated in cancer, and thus can be exploited therapeutically [95].

### Apoptosis

The mechanisms of apoptosis are highly complex and sophisticated, involving an energy-dependent cascade of molecular events. There are two main apoptotic pathways: the extrinsic or death receptor pathway and the intrinsic or mitochondrial pathway. However, these two pathways converge on the same terminal, or execution, pathway initiated by the cleavage of caspase-3 and result in DNA fragmentation, degradation of cytoskeletal and nuclear proteins, cross-linking of proteins, formation of apoptotic bodies, expression of ligands for phagocytic cell receptors and finally uptake by phagocytic cells [96]. The crucial event that commits a cell to death by the intrinsic apoptotic pathway is permeabilization of the outer mitochondrial membrane, controlled primarily by the BCL2 family of proteins, with the subsequent release of multiple proapoptotic factors that direct the physiological changes described above [97].

Within cancer cells, apoptosis is controlled by the BCL-2 family of proteins, making them powerful arbiters of cell fate in response to stress induced by neoplastic transformation as well as exposure to anti-cancer therapies [98]. Many cancers evade pro-apoptotic stress signals by up-regulating anti-apoptotic proteins such as BCL-2, BCL-xL or MCL-1 to maintain their survival; although, this may come at a cost, as these cancers may also become dependent on these anti-apoptotic proteins for survival. The development and deployment of BCL-2 family inhibitors (drugs that mimic the activity of pro-apoptotic BH3-only proteins or ‘BH3 mimetics’) is based on this paradigm, and the first potent and specific molecules are now being evaluated in clinical trials [99]. Interestingly, a first BCL-2 inhibitor (ABT-199/venetoclax), developed as a BCL-2 specific BH3-mimetic that avoids binding to BCL-xL, has been approved for the treatment of patients with chronic lymphocytic leukemia (CLL) with 17p deletion underlining the first achievement for direct targeting of the apoptotic pathway in cancer [100]. Several studies have investigated imaging probes targeting either the intrinsic or extrinsic pathway [101, 102]. Whether this will have an effect on therapy modulations still needs to be further investigated [103].

### Autophagy

Autophagy plays a key role in the maintenance of cellular homeostasis as it is a catabolic process that facilitates nutrient recycling *via* degradation of damaged organelles and proteins through lysosomal mediated degradation [104]. In neoplastic cells, autophagic responses constitute a means to cope with intracellular and environmental stress, thus favoring tumor progression; however, exerting a differential impact on malignant transformation and tumor progression [105]. Pharmacological inhibitors of autophagy exert antineoplastic effects against established tumors, especially in combination with other forms of therapy. However, highly targeted inhibitors of autophagy for use in humans are not available, and the molecules employed so far to this aim (i.e., chloroquine and hydroxychloroquine) have several therapeutically relevant off-target effects [105]. Understanding the underlying molecular mechanisms that govern these effects will allow for the development of rational approaches to manipulate autophagy for clinical benefit [106] and are under investigation [107].

### The Tumor Microenvironment: Angiogenesis / Immune cells – Fig. 2

The tumor microenvironment is increasingly recognized to play a complex role in tumor growth, development and metastases. Growth of malignant tumors requires a functional blood supply to provide nutrients, and this is facilitated and regulated by selection of pro-angiogenic peptides and growth factors in a complex interplay with regulatory anti-angiogenic factors [108].

- Angiogenesis

**Fig. 2:**
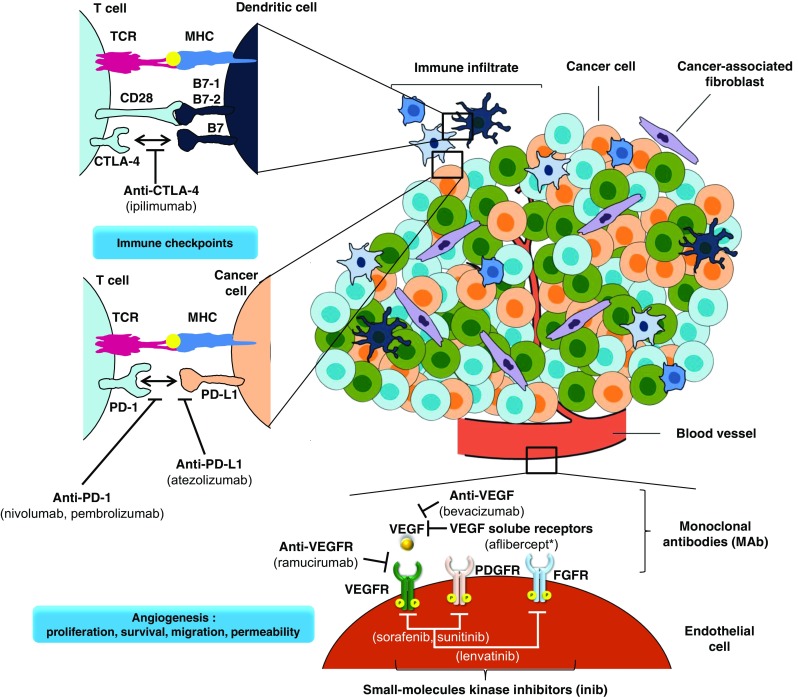
Targeting the tumor microenvironment for cancer therapy. Multiple strategies to target the tumor microenvironment are currently in clinical use as indicated here and referenced throughout the review. The tumor vasculature can be targeted with multiple drugs, such as bevacizumab (targets VEGF), aflibercept* (a soluble decoy receptor for VEGF) and sunitinib, sorafenib and lenvatinib (multi-target receptor tyrosine kinase inhibitors). Immune activation is also a promising way of therapeutic intervention which can be achieved through blockade of CTLA-4 (ipilimumab), PD1 receptor (nivolumab and pambrolizumab) or PD-L1 (atezolizumab). (*aflibercept, instead of being a monoclonal antibody, this is a synthetic fusion protein). Abbreviations: CTLA-4 (or CD152), cytotoxic T lymphocyte antigen 4; FGFR, fibroblast growth factor receptor; MHC, major histocompatibility complex; PD-1, programmed cell death 1, PD-L1, programmed cell death ligand 1; PDGFR, platelet-derived growth factor receptor; TCR, T-cell receptor; VEGF, vascular endothelial growth factor receptor; VEGFR, vascular endothelial growth factor receptor

Tumor vasculature is a field of intense study, pioneered by Judah Folkman, Bob Kerbel and Rakesh Jain [109]. Vascular Endothelial Growth Factor (VEGF) is a key driver of angiogenesis and has been recognized as an important mechanism of tumor growth, survival and metastasis in cancers [110]. VEGF overexpression has been consistently demonstrated in different cancers, and over-expression of VEGF is likely responsible for some of the pathogenomic features of advanced cancer with ascites, secondary to capillary leakiness caused by excessive VEGF [111]. It is also apparent that tumors and metastases often have disorganized internal vasculature, and unchecked cellular growth often leads to intra-tumoral hypoxia, which is a strong prognostic indicator of resistance to chemotherapy and radiation [112].

The microenvironment features of cancer growth have been incorporated into therapeutic strategies which impact upon the growth of tumors through modulation. Exploitation of this has been most notable with the incorporation of bevacizumab, a VEGF inhibitor, in conjunction with chemotherapy. Beyond VEGF, different targets have been investigated such as platelet-derived growth factor, fibroblast growth factor, angiopoietin and Ephrin type-A receptor 2 [113]. Several other VEGF targeting strategies have also led to positive results in randomized clinical trials, and have been incorporated in clinical practice. Targeting the tumor microenvironment through inhibition of tumor-associated angiogenesis has been an effective strategy in some malignancies. Currently, there are a few main approaches in targeting angiogenesis which have been tested in clinical trials and approved in clinical practice including: *i)* monoclonal antibodies binding VEGF (bevacizumab); *ii)* decoy receptors, ‘VEGF-trap’ (aflibercept); *iii)* tyrosine kinase inhibitors (sunitinib and sorafenib); and *iv)* monoclonal antibodies targeting VEGF receptors (ramucirumab) [114]. These agents are being used in the treatment of different cancer types such as breast, colorectal, hepatocellular, gastric, lung, kidney, and ovarian cancers. Clinical practice has shown that anti-angiogenic therapy is accompanied by a number of side-effects including hemorrhage, hypertension, proteinuria, impaired wound healing, thrombosis and others [115]. In many cases, antiangiogenic agents were added to standard chemotherapy and offered an improvement in therapeutic efficacy with different cancers (colorectal, non-small cell lung cancer); however, a decade after approval of the first antiangiogenic agents, antiangiogenic agents are prescribed alone as therapy for such diseases as kidney cancer or combined with different targeted therapies (i.e. lenvatinib in combination with everolimus in advanced renal cell carcinoma) [116]. Using Zirkonium-89, the *in vivo* biodistribution of bevacizumab was shown. In another study the modulation of the hypoxic area within a tumor by bevacizumab was demonstrated by means of a hypoxia PET tracer [17, 117, 118].

- Immune Modulation

The importance of intact immune surveillance in controlling outgrowth of neoplastic transformation has been demonstrated in preclinical models [119]. Accumulating evidence shows a correlation between tumor-infiltrating lymphocytes (TILs) in cancer tissue and favorable prognosis in various malignancies. In particular, the presence of CD8+ T-cells and the ratio of CD8+ effector T-cells / FoxP3+ to regulatory T-cells correlates with improved prognosis and long-term survival in many solid tumors [120].

Immunotherapy is defined as the approach to treating cancer by generating or augmenting an immune response against it [4]. Two types of immunotherapy have emerged as particularly effective over the past decade: immune-cell-targeted monoclonal antibody (mAb) therapy and adoptive cellular therapy [121]. Since the first approval of a checkpoint inhibitor (ipilimumab) as monotherapy for the treatment of advanced melanoma, several immune-checkpoint-blocking mAbs - cytotoxic T lymphocyte-associated protein 4 (CTLA-4) and programmed cell death protein 1 (PD1) - have been approved for the treatment of patients with several types of cancer (Fig. 2).

One approach to augment antitumor immune responses has been termed “checkpoint blockade”. This term refers to the strategy that targets the natural negative signals that are used to regulate the immune response [122]. T cell activation is regulated by a variety of activating and inhibitory receptor/ligand interactions [123]. One of the well-studied inhibitory molecules is PD-1, which is expressed on the cell surface of activated T-cells [124–126]. PD-1 (encoded by the gene *Pdcd1*) is an immunoglobulin (Ig) superfamily member related to CD28 and CTLA-4, which negatively regulates antigen receptor signaling upon engagement of its ligands (PD-L1 and/or PD-L2) [127]. PD-1 and family members are type I transmembrane glycoproteins containing an Ig Variable-type (V-type) domain responsible for ligand binding and a cytoplasmic tail that is responsible for the binding of signaling molecules. PD-1 has been shown to be expressed on activated lymphocytes including peripheral CD4+ and CD8+ T-cells, B-cells, T regs and Natural Killer cells [128]. The ligands for PD-1 (PD-L1 and PD-L2) are constitutively expressed or can be induced in a variety of cell types, including non-hematopoietic tissues as well as in various tumors. Binding of either PD-1 ligand to PD-1 inhibits T-cell activation triggered through the T-cell receptor. PD-L1 is expressed at low levels on various non-hematopoietic tissues, most notably on vascular endothelium, whereas PD-L2 protein is only detectably expressed on antigen presenting cells found in lymphoid tissue or chronic inflammatory environments. PD-L2 is thought to control immune T-cell activation in lymphoid organs, whereas PD-L1 serves to dampen unwarranted T-cell function in peripheral tissues. Although healthy organs express little (if any) PD-L1, a variety of cancers were demonstrated to express this T-cell inhibitor. Recent studies suggest that PD-L1 is upregulated only when tumor cells are in close proximity with T cells in the tumor microenvironment [129–131]. The PD-1/PD-L1 pathway may play a critical role in tumor immune evasion and can be considered as an attractive target for therapeutic intervention in specific cancers. Indeed, PD-1 inhibitors, such as nivolumab and pembrolizumab have been approved in melanoma and non-small cell lung cancers (NSCLC). Very recently, a PD-L1 antibody (atezolizumab) was approved for the treatment of patients with metastatic NSCLC who have progressed on prior chemotherapy (and targeted therapy, for those with EGFR or ALK genetic alterations) showing that novel immunotherapies are playing an increasing role in the treatment of NSCLC [132]. PD-1 and PD-L1 checkpoint inhibitors are associated with a specific spectrum of immune-related adverse events with potential dermatological, gastrointestinal, pulmonary, endocrine, renal and hepatic toxicities. This spectrum is different from toxicities known for kinase inhibitors or cytotoxic drugs and are, in general, reversible and manageable with immunosuppressive therapy, which is indicated for utmost all events of moderate to serious severity [131]. Others strategies are under evaluation including combination treatment and oncolytic virus therapy [4]. Several approaches have already been investigated to implement targeted imaging of PD-L1 expression [133–135].

As human cancers carry a multitude of somatic gene mutations and epigenetically altered genes, the products of which are potentially recognizable as foreign antigens, immunotherapy seems an attractive strategy. PD-1 blockade may enhance the immune response to these mutated antigens. The neoantigen load may form a biomarker in cancer immunotherapy and provide an incentive for the development of novel therapeutic approaches that selectively enhance T cell reactivity against this class of antigens [136].

### Targeted Therapy – Challenge of Imaging in Cancer

In the field of oncology, there continues to be a strong drive towards advancing and achieving personalized medicine, such that treatments are tailored to the individual patient. Based on the mechanism of drug action, imaging assessment is evolving in parallel. This contemporary challenge will persist since a large number of drugs are developed in each of the three systemic therapeutic modalities: classical cytotoxics, new targeted agents, and emergent immunotherapeutic approaches. Ideally, tumor and patient evaluations will lead to the selection of the best treatment (based on tumor characterization) and the right dosing schedule (based on patient characterization) [137]. Although our understanding of the molecular complexity of cancer has increased over the years, many challenges remain including disease heterogeneity, clinical and genomic patient variability, limited number of effective treatments, and drug delivery. Thus, it is imperative to devise innovative and adaptive imaging assessment to accelerate our efforts in improving diagnosis, disease extension and therapy efficacy.

Another consideration in terms of imaging perspective is knowledge of drug mechanics, drug chemical (TKI, Antibody) and drug half-life for imaging schedule [138]. Indeed, molecular therapies that either have a short life or target pathways exhibiting recovery early after drug cessation may interfere in the result interpretation of the conventional FDG PET for response assessment [139]. The delay between the last drug intake could lead to inaccurate results if a patient was scanned several days after treatment cessation, at a time when target inhibition is no longer present. The drug schedule/interruption may have been determined by the prescription of the referring oncologist, but also by poor observation or recording of medication. Recording of the last drug intake before PET scanning is not routinely done in PET units, though this can impact the results of imaging, particularly for therapies with short half-life. The drug pharmacokinetics should be considered to establish the PET imaging time points. Given the potential chronic administration and the increased prescription of oral target therapies, compliance to drug intake may be compromised and, as such, there is a need to systematically incorporate into the PET reports information regarding the date and time of the last drug intake.

These new therapies require novel tools and potential targeting imaging to assess the response to treatment [140, 141]. Pseudoprogression, tumor growth from treatment effect rather than true disease progression, has been described with immune checkpoint inhibitors. Pseudoprogression is uncommon and indicates a high likelihood of > 1 year survival and, as such, needs to be identified [142]. These novel agents entail a whole new series of concepts that have resulted in a number of opportunities for novel imaging tools. Such innovation may be incorporated in trials and would allow for better selection of candidate populations, discovering and validating biomarkers, defining suitable endpoints, and proposing increasingly more accurate non-invasive imaging response criteria, including radiological as well as molecular imaging information [143, 144].

Targeted imaging beyond classical FDG has evolved in the last years very rapidly; however, the fact that every new imaging probe has to undergo the same steps as a new pharmaceutical has hampered the broad implementation of new radiopharmaceuticals. The knowledge of biodistribution aspects of different probes is of great importance. Furthermore the right radionuclide should be used to be able to follow, for example, the long biological half-life of antibodies.

## Conclusion

Staging in the era of precision and personalized therapy (target driven) has created an exciting new platform of development for imaging. The ‘classic’ FDG PET-scan will improve to a personalized PET-based molecular imaging [145], which will result in parallel improvements in response prediction [146], therapy monitoring [147, 148], potential pharmacodynamic markers and quantitative imaging clinical trials [149]. Our understanding of the evoked cellular processes in response to DNA damage has improved considerably in recent years. Advancements in this area have revealed attractive biomarkers, which could be used to improve upon existing methods for non-invasive early cancer detection and therapy evaluation. A range of PET/SPECT imaging agents are currently under development and poised for evaluation in the clinical setting.
